# Challenges in the assessment of nursing students in clinical placements: Exploring perceptions among nurse mentors

**DOI:** 10.1002/nop2.717

**Published:** 2020-11-27

**Authors:** Bjørg Christiansen, Gertrud Averlid, Cynthia Baluyot, Karin Blomberg, Anne Eikeland, Ingrid Rachel Strand Finstad, Monica Holm Larsen, Katrin Lindeflaten

**Affiliations:** ^1^ Department of Nursing and Health Promotion Faculty of Health Sciences Oslo Metropolitan University Oslo Norway

**Keywords:** clinical placement, mentorship, nurses, nursing

## Abstract

**Aim:**

The aim was to explore how nurse mentors experience the assessment of nursing students in clinical placements at hospitals and in municipal health care.

**Design:**

The study is qualitative with an explorative and descriptive design.

**Methods:**

Based on an interview guide, we conducted 19 individual qualitative interviews and four focus group interviews with nurse mentors from various levels and fields of nursing education at a Norwegian university.

**Results:**

Feedback in and on action was an integrated part of the formative assessment. In the summative assessment, where the university lecturer also participates, the nurse mentors perceived their role as passive. A disturbing finding was that divergent views on the student's competence sometimes occurred in these situations, thus challenging the credibility of the student assessment. Perceptions of nursing values and concerns embedded in nursing practice as collective criteria appear to have an impact on the mentors’ assessment of the nursing students.

## 1 INTRODUCTION

The assessment of competence within a learning environment is a key element of nurse mentors’ supervision of nursing students during their clinical placements. An essential part of the assessment is to provide feedback to the student. According to Boud (2015), assessment and feedback that influence learning require knowledge of appropriate standards, comparison of the work performed with these standards and taking of action to close the gap between the two. A distinction is usually made between a continuous formative assessment considered to be supportive for learning and a final summative assessment, which concludes whether the student's overall competences are in accordance with the programme's expected learning outcomes (Vinales, 2015). Placements are often assessed by means of a pass or fail rather than awarded a grade (Heaslip & Scammel, 2012).

Formative feedback is ongoing and aims to improve the learning experience. Feedback should, according to Clynes and Raftery (2008), provide the student with information on current practice and offer practical advice for improved performance. Previous research shows that effective, critical and constructive feedback as part of continuous assessment enables nursing students to identify their strengths and weaknesses (Adamson et al., 2018; Clynes & Raftery, 2008). Nurse mentors assess the students’ attitudes, knowledge and skills within a clinical learning environment. Evaluating the students’ performance of nursing is therefore a complex activity that involves identifying how each student's competences are expressed in various clinical situations and giving feedback in the light of professional criteria, usually defined in assessment tools. Learning outcomes are assertions about the results of learning in an educational activity, often defined in terms of a mixture of knowledge, skills, abilities, attitudes and understanding (Adam, 2008). However, earlier research into the assessment of nursing students during placements shows that there are challenges concerning the general or difficult language used in assessment tools (Helminen et al., 2016; Wu et al., 2015).

In Norway, nurse mentors have the main responsibility for the continuous assessment process of the nursing students. As such, they also contribute to providing a valid summative assessment, entailing a great responsibility for ensuring that those taking the education are sufficiently qualified. Nurse mentors are clinical nurses, of whom some have attended a three‐day course in supervision and assessment of nursing students at the university. The university lecturer supervises the students in groups on themes and assignments related to the placement, usually four to five times in the course of the eight weeks of placement. Another important role of the university lecturer is to support the nurse mentor in performing appropriate assessments, especially in relation to weaker students. The midterm and final summative assessments take the form of a triangular conversation between the student, nurse mentor and university lecturer. These meetings are agreed in advance in the course of the placement. The university lecturer, on behalf of the nursing education programme, has the formal responsibility for the midterm and final assessment and often chairs the meetings (Bachelor's Programme in Nursing, 2019).

Although several challenges concerning the assessment of nursing students during clinical placements have been reported earlier, not least concerning the use of assessment forms (Helminen et al., 2016; Wu et al., 2015), there is a lack of studies that investigate the characteristics of the formative and summative assessment of students from the perspective of nurse mentors.

## 2 BACKGROUND

### 2.1 Learning outcomes as criteria in assessment

The shift towards the use of learning outcomes has led to a greater understanding of the central importance of assessment and feedback in learning processes (Havnes & Prøitz, 2016). Learning outcomes were originally associated with the Bologna Process and have an impact on all sectors of European education (Adam, 2008). In Norway, learning outcomes were introduced in 2011 and apply to all levels of education. Each nursing education programme has therefore developed a curriculum where learning outcomes provide direction for learning and assessment in theoretical and practical topics, which is also reflected in the assessment forms or tools used during clinical placements. In Sweden, Löfmark and Thorell‐Ekstrand (2014) developed an assessment form, which in its current form is called AssCE (Assessment of Clinical Education). The nursing education programme at Oslo Metropolitan University (OsloMet) uses a simplified version of this form. Learning outcomes as criteria for assessment are still relatively generally defined and can therefore entail challenges in the assessment process in clinical placements.

Various challenges concerning the use of assessment tools are reflected in international studies in the field of nursing education. In a review article, Wu et al. (2015) claim that differences in the lecturers’ and preceptors’ interpretation of the assessment tool had an impact on the reliability of the assessment. Other findings also indicate differing views on assessment processes. Mentors and teachers have stated that honest and direct criteria‐based final assessment was carried out more often than the nursing students believed (Helminen et al., 2017). Similar findings in a review article suggest that the process of assessing nursing students’ competences in clinical placements lacks consistency. The terminology in the assessment forms is sometimes so difficult to grasp that the mentors do not fully understand what the various points mean. The quality of assessment varies greatly and is also open to the subjective bias of the assessor (Helminen et al., 2016).

Some of the criticisms of learning outcome descriptions claim that they are usually defined in a way that takes little account of social and contextual aspects of learning. According to Havnes and Prøitz (2016), if students’ learning is unilaterally governed by predefined standards, a learning potential can be lost. In their literature study with emphasis on the relationship between learning views and learning outcome descriptions, the authors demand an open, process‐oriented approach to learning outcomes in educational institutions. This means recognizing contextual variations in the assessment and documentation of learning, which also includes the unintended and contingent in learning, not just what is predefined and measurable (Ibid.).

### 2.2 Assessment in a learning environment

From a socially situated perspective, learning is understood with reference to the context where persons act (Lave & Wenger, 2003). Wimmers and Mentkowski (2016) therefore argue that learning in the profession is best understood as a process embedded in social relationships and social practices, for example with other learners, professionals and patients. The authors also underline the importance of linking the assessment of competences to criteria that are determined by the contexts of practice, or to what a learner does with what she/he knows, in context (Ibid.). Thus, the continuous assessment process enables a valid impression of how the students’ competences manifest themselves in various clinical situations over time.

Clinical situations (e.g. helping a patient with morning care routines) constitute a learning environment for the student where learning outcomes and behavioural cues are contextually rooted: To be able to understand patients’ situations and adopt a moral and professional responsibility for their well‐being, nursing students must develop awareness of the patients’ reactions and emotional states as an ongoing skill, even beyond what they express verbally. The assessment process may take place as context‐dependent deliberations, for example whether the student is able to establish a trusting relationship, whether they take into account that the situation could cause pain, or whether they show manual dexterity. Thus, a clinical situation may facilitate a comprehensive assessment that applies to several learning outcomes.

Although the student is the protagonist of all assessment situations, the power relationship is uneven, particularly when it comes to the midterm and final assessments. The summative character of the midterm assessment is exacerbated by the process of filling out the assessment form as well as forming conclusions with regard to whether the student's competences so far are “as expected” or not and what she/he ought to work on further during the placement. The students normally self‐assess their performance, followed by an assessment given by the university lecturer and the nurse mentor. The university lecturer's assessment is usually based on their impressions of the student from group meetings and written study assignments related to the clinical placement. If the conclusion at the midterm assessment is that the student's competences so far are below the expected level, extra follow‐up is usually planned. According to Helminen et al. (2016), few studies have specifically explored the phenomenon of summative assessment.

Proper assessment of nursing students is not only an important tool in learning processes, but also a means to ensure patient safety. In this endeavour, the nurse mentors play an important role. However, the status of knowledge shows that there is a need for further research into the nurse mentor's role in the assessment of nursing students’ competences during clinical placements. The aim of this study was thus to explore how nurse mentors experience the assessment of nursing students in clinical placements at hospitals and in municipal health care.

### 2.3 Research questions

- How do nurse mentors perceive the use of the assessment form?
- How do nurse mentors experience the continuous assessment process of students’ competences?
- How do nurse mentors experience the midterm and final assessment?

## 3 METHODS

### 3.1 Design

The study is qualitative with an explorative and descriptive design. Qualitative interviews are well‐suited to providing insight into how nurse mentors perceive the assessment of nursing students in clinical placements (Brinkmann & Kvale, 2015). A group of eight faculty researchers/teachers in the research group *Learning and Interaction* at the Department of Nursing and Health Promotion at OsloMet in Oslo conducted the study in autumn 2017 and spring 2018 (Table 1).

**Table 1:** Participants

Levels of nursing education	Fields of nursing education	Participants (nurse mentors)	Qualitative interviews
Anaesthesia and intensive care, master level (MA)	Anaesthesia and intensive care wards (AIW)	7 (4 intensive care, 3 anaesthesia)	Individual interviews
Third year bachelor (BA)	Medical and surgical wards (MSW)	6	Individual interviews
Second year BA	Home care nursing (HCN)	11	3 focus group interviews
Second year BA	Psychiatric wards (PW)	6	Individual interviews
First year BA	Nursing home (NH)	3	1 focus group interview

### 3.2 Participants and recruitment

A convenience sample of 33 nurse mentors from various levels and fields of nursing education were recruited to participate in either individual or focus group interviews. Our sampling strategy involved asking the nurse manager at each ward to recruit participants who had at least one year's experience as a nurse mentor for our students. Their experience with supervision and assessment of nursing students varied from one–25 years. Some of them had attended a three‐day course in supervision and assessment of students at the university. However, this was not an inclusion criterion for participating in the study. The researchers provided further written and oral information about the study.

### 3.3 Data collection

Based on an interview guide, we conducted 19 individual qualitative interviews and four focus group interviews with the nurse mentors. The semi‐structured interview guide was based on themes agreed in the research group and adjusted to the various levels and fields of the education. The interview guide covered the following themes:

- Characteristics of the learning environment at the clinical placement
- Experiences from the formative assessment process
- Perceptions of the assessment form
- Experiences from the midterm and final assessment

The focus group interviews required a more open, thematic guide, while some sub‐questions were added to the semi‐structured guide used in the individual interviews. We considered that both forms of qualitative interviews were suitable for data collection in this study, because both discussions as well as detailed, individual responses could contribute to providing variation in the empirical material. Thus, the type of interview was decided by the researchers in cooperation with the leaders and participants from each ward.

Individual qualitative interviews are well‐suited to providing insight into themes from the patient's own perspectives (Brinkmann & Kvale, 2015). Each participant decided where the interview should be carried out, in the ward or at the university. We sought to encourage the participants to voice their experiences with assessing nursing students in their own terms, while also ensuring that the themes in the interview guide were covered. The interviews lasted from 45–60 min.

The aim of a focus group interview is to use group interaction to obtain the participants’ views, experiences and opinions on the patient in question (Brinkmann & Kvale, 2015; Krueger & Casey, 2009). The participants were asked to exemplify and discuss how they experienced the assessment of nursing students. The focus group interviews were conducted in the wards by two researchers, where one acted as a moderator and the other as co‐moderator who contributed with follow‐up questions. The focus group interviews lasted from 40–60 min. All interviews were recorded and transcribed verbatim by a research assistant.

### 3.4 Data analyses

Although the interviews from the various fields of nursing education were initially analysed separately by the researchers, the research group employed a common strategy in cooperation with the manager of the project (first author). The analysis of the transcriptions from the interviews was inspired by Brinkmann and Kvale (2015) and Braun and Clarke’s (2006) approaches to qualitative analysis and was carried out using various tools. This included coding and categorization of meanings on three levels:

First level: After performing in‐depth readings of the transcriptions to gain a sense of the overall picture, meaning units that were derived from the data were identified by colour‐coding to structure the participants’ utterances. The transcriptions from each field of nursing education were searched for similar and contrasting utterances and condensed into preliminary themes (e.g. perceptions of the assessment form, characteristics of the learning environment, expectations of the students, characteristics of feedback and cooperation with the lecturer). According to Brinkmann and Kvale (2015), this level of interpretation is confined to the patient's self‐understanding.

Second level: In the further attentive reading and discussions of the material, we focused on the research questions. The content of the preliminary themes was compared and merged into four themes, including a wider frame of understanding than that of the participants themselves (Brinkmann & Kvale, 2015). To enhance the rigour of our interpretations, another three researchers from the research group were involved in the analytic process, which opened up for more nuanced meanings. Through this process, the content of the four main themes eventually emerged. Verbatim quotations from the transcribed material underpinned and exemplified our interpretations as reflected in the presentation of results: A gap between learning outcomes and the learning environment; the characteristics of the nurse mentors’ assessment; context‐dependent feedback; and a passive role in the midterm and final assessment.

Third level: As reflected in the discussion, this is a more comprehensive interpretation where our theoretical framework and previous research move our analysis to a higher level of abstraction (Brinkmann & Kvale, 2015).

### 3.5 Methodological considerations

One challenge in the data collection process and further analysis was that the researchers also had experience as teachers at some of the wards. According to Rubin and Rubin (2012), it is an advantage to have knowledge of the culture being studied (as a teacher), whereas the challenge is to create an analytical distance from the taken‐for‐granted knowledge. For this reason, we tried to avoid a situation where the researchers and the participants knew each other beforehand.

Concerns about validity were attended to by conducting interviews with nurse mentors from various levels of nursing education, even though these settings do not represent the full spectrum of placements. Although it may be considered a methodological challenge that we obtained data from both individual and focus group interviews, emphasis was placed on allowing the participants to talk freely and matter‐of‐factly on the themes in focus in all of the interviews. Despite using the same themes in the interview guide, the fact that we were a group of researchers may have influenced the course of each interview to some degree. Ongoing dialogue with the research manager and discussions with other researchers in the research group enriched our analysis. According to Brinkmann and Kvale (2015), different interpreters can be a source of fruitfulness and virtue in interview research. It would be of interest to conduct further empirical work into how assessments are performed, including their significance for learning.

## 4 RESULTS

### 4.1 A mismatch between learning outcomes and the learning environment

An overarching result was that the nurse mentors from the various levels of nursing education perceived a mismatch between the learning outcomes given in the assessment form and the learning environment. Because the learning outcomes to a lesser degree complied to distinctive, contextualized features of nursing, their relevance as criteria for assessing the students’ competences were impaired: The mentors from the anaesthesia and intensive care wards perceived the learning outcomes in the assessment form to be “indefinite and overwhelming” (AI, master). In nursing homes, the nurse mentors felt that the general formulations used in the assessment form gave few concrete guidelines for their assessment of the students’ competences in the learning environment (NH, 1st year). However, some mentors from medical and surgical hospital wards appreciated the general formulations in the assessment form, because it gave them an opportunity to adapt the learning outcomes to the particular placement (MSW, 3rd year). On the other hand, nurse mentors in home care nursing felt that there was a lack of more realistic learning outcomes that embraced the distinctiveness of the placement. The style of language used in the assessment form was, according to them, unclear and insufficiently concretized: “What does a patient phenomenon mean?” (HCN, 2nd year). Nurse mentors from psychiatric wards also perceived the language in the assessment form as “very theoretical”. To compensate, some of them had employed self‐made assessment tools “based on the assessment form and what is special about this placement” (PW, 2nd year).

### 4.2 Characteristics of the nurse mentors’ assessment

Results show various examples of how the assessment process was influenced by the nurse mentors’ expectations of student behaviour, as well as features of the learning environment: The nurse mentors from nursing homes expected the first year students to take responsibility for their own learning. They soon became aware of passive students: “Just standing and not talking to the patient…or not trying to do anything” (NH, 1st year). They agreed that this was usually due to insecurity in the initial phase of the placement and tried to support and even defend the student to colleagues in the ward who had a negative first impression of them. However, the results also show that their assessment could be based on personal expectations of good nursing: “Would I have liked to be cared for by this student? We often base the assessment on our own values and attitudes, how we ourselves would have liked it to be” (NH, 1st year). There were also examples of how the assessment process involved the opinion of others: If the nurse mentors from home care nursing were insecure about a student's competency, they often consulted each other: “Can you see if you perceive her/him the way I do?” (HCN, 2nd year). Thus, they perceived their colleagues to be an important resource to ensure a fair and justifiable assessment.

Particular challenges in the learning environment influenced their expectations of the students’ competences: At a paediatric department, the nurse mentors assessed the third year students’ ability to communicate in complex situations in the ward, even if this was not a given criterion in the assessment form: “To adapt to children of different ages”, not least when there is “an uncertain course of their illness” (MSW, 3rd year). Nurse mentors from psychiatric wards found it challenging to assess the second year students because there were few clinical procedures and the focus in the ward was primarily on “personal behaviour and the ability to exercise discretion” (PW, 2nd year). There seems to be agreement that the placement gave the students an opportunity to develop interpersonal qualities, for example: “to distinguish between being private and professional in relations with patients” (PW, 2nd year). Nurse mentors from anaesthesia and intensive care wards emphasized asking questions to gain insight into “how the students perceive the patient's situation or the implementation of procedures”. The students had to verbalize their understanding relative to ongoing learning situations, “so we understand that they understand” (AIW, master).

### 4.3 Context‐dependent feedback

According to the nurse mentors, their feedback focused on student behaviour and understanding in specific situations: In nursing homes, feedback was often related to the course of action, for example when wound‐dressing: “Why are you doing that?” (…) “We usually correct them in a proper way in‐action, unless there is a situation where the patient should not hear it”. If ongoing feedback was improper, they assessed the student's performance and alternative course of action together afterwards (NH, 1st year). Nurse mentors from psychiatric wards emphasized giving feedback on the way the students positioned themselves and communicated, both verbally and non‐verbally, with vulnerable patients in the ward: “What do you signal if you (the student) are biting your nails in conversation with someone?” They often recommended that the students “read up on relevant theory” concerning the patients at the ward (PW, 2nd year).

The nurse mentors in home care nursing emphasized time pressure as a hindrance to the supervision and assessment of second year students: “I feel we don't have time to talk to the students every day, except in the car before we get to the user” (HCN, 2nd year). Nurse mentors from medical and surgical wards often felt insecure in their assessment and feedback regarding commitment and knowledge among third year students. Some mentors found it difficult to communicate weaknesses in the student's competence, especially if the student was “weak in action, but verbally strong “(MSW, 3rd year). In such situations, they often felt alone with the responsibility for assessment and feedback and missed having more collaboration with the university lecturer.

### 4.4 Passive role in the midterm and final assessment

In the midterm and final assessment situations, the university lecturer also participated and chaired the meetings. Usually, the students assessed themselves initially by going through the learning outcomes in the assessment form. The nurse mentors had often prepared themselves beforehand, as this example from a nursing home shows: “ I sit at home or at work and fill it out (the assessment form) and recall special situations with patients or practical things (…) so I can fill in or underpin things (during the meeting)” (NH, 1st year). Even if their opinion was asked for, most of the nurse mentors from various levels of nursing education experienced a more or less passive role in these meetings, in some cases also with a feeling of being assessed along with the student:

It is the teacher who chairs the meeting. I feel a bit left out; the assessment is something going on between student and lecturer (…) But if the student needs help, I contribute with input. (HCN, 2nd year)

Although the nurse mentors from the anaesthesia and intensive care wards also seemed to have a passive role during the final assessment, they often perceived a consensus of opinions: “ I just confirm what is being said (…) If the lecturer asks for something then we can supplement, confirm or disagree” (AI, master). Thus, the midway and final assessment situations were perceived as formal, even though these meetings were at times conducted as more of a conversation than an interview (PW, 2nd year).

Some mentors found that the university lecturer focused too much attention on the study assignment (PW, 2nd year). Their opinions of the students’ competence could also diverge: “What the lecturer saw in the paper (the study assignment) was an A grade, whilst what I saw in practice was a D grade” (MSW, 3rd year). This nurse mentor worried that different expectations of the students’ competences could affect their credibility in the eyes of the student. However, the lecturers’ credibility in assessing the students’ competences varied in the eyes of the nurse mentors: “No offence to the lecturer, but it's been a few years since they worked in the clinical field” (AI, master). To get a better grasp of the relationship between learning outcomes and opportunities in the learning environment, several of the nurse mentors expressed the wish that the lecturer had more up‐to‐date knowledge of clinical practice (MSW, 3rd year). Another common result was that the nurse mentors wanted more regular contact with the university lecturer during the clinical placement, particularly if they were uncertain about a students’ competency. They also wanted to be informed about the patient s that the lecturers and students worked on in the group meetings.

## 5 DISCUSSION

The nurse mentors found it challenging to see how the expected learning outcomes in the assessment form complied with characteristics of nursing in the clinical environment. Because they perceived the language in the assessment form as general and theoretical, the value of expected learning outcomes as criteria for their assessment of the students’ competences was impaired. Other studies have also shown great challenges concerning the implementation of assessment forms, either to find relevant clinical situations to exemplify and concretize the content (Mårtensson et al., 2020), or to understand the terminology and level of concretization in the assessment forms (Helminen et al., 2016; Wu et al., 2015).

Our study shows examples of how the nurse mentors’ expectations of the students’ behaviour coloured their assessment. According to Helminen et al. (2016), assessments are also open to the subjective bias of the assessor. Results show that the nurse mentors did not solely rely on their subjectivity, but also incorporated the opinions of their colleagues, referring to what they, or “we”, consider nursing standards in the ward. Vinales (2015) suggests that other experienced practitioners should be involved in the assessment of pre‐registration students, as this would limit bias and can ensure transparency and fairness across assessments. Although the assessment process involves identifying appropriate standards and criteria (Boud, 2015), criteria for good work and nursing expertise may also be tacitly agreed on among colleagues (Benner et al., 2009; Gardner et al., 2001). Thus, spoken and unspoken ideal perceptions of nursing values and concerns are embedded in nursing practice as collective criteria, which also have an impact on the assessment of nursing students. However, as the results indicate, the coherence with defined learning outcomes is easily blurred, influencing the transparency and reliability of the assessment.

An overall result was that the process of assessment was contextually rooted in the learning environment at the placement. When a new first year student was perceived as “passive” and reluctant in relation to patients, the nurse mentors searched for context‐depended explanations, such as insecurity when facing challenges in nursing homes. Students’ relational competences were developed and assessed in clinical situations, which required a personal and professional approach, for example interacting with seriously ill children or with psychiatric patients. Thus, the assessment of student behaviour was always situated, referring to the student's performance in clinical situations, thereby providing a context for learning and assessment. Dreyfus and Dreyfus (1980) claim that a critical context‐dependent assessment can only be acquired in live clinical practice. Wimmers and Mentkowski (2016) also underline that assessments of competences must be linked to criteria which are determined by the contexts of practice.

There are, however, few examples of assessments of professional reasoning along with observable behaviour in our results, with the exception of placements for master level students, where the nurse mentors often asked for the students’ reasoning in connection with them performing procedures. The concept of “practical synthesis” means that theoretical content can only be synthesized when directed towards and integrated in the performance of practical tasks (Heggen & Terum, 2013).

Our study shows that feedback was an integrated part of the assessment process, focusing on students’ behaviour and understanding in specific situations, both in and after action. As the results indicate, feedback is obviously a greater challenge when the nurse mentor discovered a gap between “saying and doing” concerning a student's exercise of nursing. When facing particular challenges with student feedback, they wanted better collaboration with the university lecturer. Although the importance of feedback in clinical practice is widely acknowledged, it appears that there is inconsistency in its provision to students (Clynes & Raftery, 2008; Henderson et al., 2012).

The midterm and summative assessments have a more formal character. An overall result was that the nurse mentors experienced their role as passive in these settings, even though their opinion was asked for by the university lecturer. When going through the learning outcomes in the assessment form, divergent views on the student's competences sometimes occurred, challenging the credibility of the assessment of the student. This is disturbing because apart from providing feedback, the summative assessment also includes grading the student's clinical performance (Helminen et al., 2016).

Because the provision of the nurse mentors’ assessment is paramount, our study shows a need for more transparency and mutual understanding of the assessment of nursing students during practical placements. One way to achieve this is in the form of a local curriculum that contextualizes the defined learning outcomes in a clinical learning environment. To secure coherence and usability as a tool for learning and assessment, it is of importance that a local curriculum is developed by representatives from both the clinical placement (nurse mentors) and the nursing education programme (university lecturers).

## 6 CONCLUSION

The study shows that the nurse mentors found it challenging to use defined learning outcomes as criteria for their assessment of the students’ competences, particularly due to the general language used in the assessment tool. Thus, perceptions of nursing values and concerns embedded in nursing practice as collective criteria seem to have an impact on their assessment of the nursing students. Moreover, the study shows few examples of assessing professional reasoning along with observable behaviour. This is an issue that needs to be further investigated. Feedback in and on action was an integrated part of the assessment process, although when facing particular challenges, the nurse mentors wanted better collaboration with the university lecturer. In the summative assessment, where the university lecturer also participated, the nurse mentors experienced their role as passive. A disturbing result was that divergent views on the students’ competences sometimes occurred in these situations, challenging the credibility of the assessment of the students.

## CONFLICT OF INTEREST

The authors declare no conflict of interest.

## AUTHORS’ CONTRIBUTION

All authors fulfil the journal's authorship policy, and have approved the final article text. All authors have made substantial contributions to conception and design, acquisition and analysis of data. First author assumes the main responsibility for the manuscript preparation, but all authors revised the content critically.

## ETHICAL APPROVAL

The study was approved by NSD (The Norwegian Data Inspectorate). Permission to recruit nurse mentors was given by the head nurse of each ward. Participants who took part in the interviews received written information and signed consent forms. They were informed that participation was voluntary and that they had the right to withdraw at any time. In the following presentation of data, no identifying characteristics are used.

## Data Availability

To access the data in this study, please contact the corresponding author.
